# Combined roles of exporters in acetic acid tolerance in *Saccharomyces cerevisiae*

**DOI:** 10.1186/s13068-022-02164-4

**Published:** 2022-06-18

**Authors:** Xiaohuan Zhang, Jeroen G. Nijland, Arnold J. M. Driessen

**Affiliations:** Molecular Microbiology, Groningen Biomolecular Sciences and Biotechnology, University of Groningen, Nijenborgh 7, 9747 AG Groningen, Netherlands

**Keywords:** Acetic acid, Acetate efflux, Aqr1, Tpo2, Tpo3, *Saccharomyces cerevisiae*

## Abstract

Acetic acid is a growth inhibitor generated during alcoholic fermentation and pretreatment of lignocellulosic biomass, a major feedstock to produce bioethanol. An understanding of the acetic acid tolerance mechanisms is pivotal for the industrial production of bioethanol. One of the mechanisms for acetic acid tolerance is transporter-mediated secretion where individual transporters have been implicated. Here, we deleted the transporters Aqr1, Tpo2, and Tpo3, in various combinations, to investigate their combined role in acetic acid tolerance. Single transporter deletions did not impact the tolerance at mild acetic acid stress (20 mM), but at severe stress (50 mM) growth was decreased or impaired. Tpo2 plays a crucial role in acetic acid tolerance, while the *AQR1* deletion has a least effect on growth and acetate efflux. Deletion of both Tpo2 and Tpo3 enhanced the severe growth defects at 20 mM acetic acid concomitantly with a reduced rate of acetate secretion, while *TPO2* and/or* TPO3* overexpression in *∆tpo2∆tpo3∆* restored the tolerance. In the deletion strains, the acetate derived from sugar metabolism accumulated intracellularly, while gene transcription analysis suggests that under these conditions, ethanol metabolism is activated while acetic acid production is reduced. The data demonstrate that Tpo2 and Tpo3 together fulfill an important role in acetate efflux and the acetic acid response.

**Supplementary Information:**

The online version contains supplementary material available at 10.1186/s13068-022-02164-4.

## Introduction

Lignocellulosic biomass is widely and abundantly present on earth and is considered a second-generation feedstock to produce bioethanol and other building block chemicals. The advantage of lignocellulosic biomass as a feedstock is that it does not compete with food crops. Pretreatment of lignocellulose is crucial for utilization. One of the by-products in this process is acetic acid, an inhibitor that hinders the growth of the yeast *Saccharomyces cerevisiae* and that in particular interferes with pentose sugar fermentation [13]. Additionally, acetic acid is also produced during alcoholic fermentation [18]. The response of *S. cerevisiae* to an acetic acid challenge is complex and diverse. Amongst others, this results in the accumulation of reactive oxygen species (ROS) and subsequently the release of cytochrome *c* which activates Yca1, a Ca^2+^-dependent cysteine protease which is homologous to caspases and that removes insoluble protein aggregates while causing apoptosis [12, 20]. ROS-independent acetic acid-induced programmed cell death (AA-PCD) has also been observed and likely involves ceramides [11].

The cellular toxicity of acetic acid largely depends on the environmental pH. When the pH is below the pKa (i.e., pH 4.76) of acetic acid, the undissociated form of acetic acid readily crosses the cytoplasmic membrane by passive diffusion or via the aquaglyceroporin protein Fps1 [24]. The acid dissociates inside the cells thereby lowering the intracellular pH. As a response, the H^+^-ATPase Pma1 pumps out the protons in order to maintain a stable intracellular pH. On the other hand, the dissociated acid, acetate, is exported by transporters, like Tpo2 and Tpo3 [29]. Additionally, the transporter Aqr1 was reported to confer resistance to acetic acid [36]. Acetate stress is able to cause the activation of Hog1, the mitogen-activated protein (MAP) kinase, and of the transcriptional activator Haa1 [14, 21, 22, 24]. Hog1 destabilizes Fps1 thereby eliminating the major route for acetic acid influx in to the cell [24], while Haa1 regulates gene transcription involved in stress adaptation [10]. At increasing acetic acid concentrations, the phosphorylation of Haa1 is slightly increased, which is negatively regulated by the casein kinase I isoform Hrr25 [4]. Consequently, this activates Haa1 regulated gene transcription. The translocation of Haa1 from the cytoplasm to the nucleus, which is likely determined by the degree of Haa1 phosphorylation, is necessary for the transcription of Haa1-targeted genes [4]. The Haa1-activated gene response towards acetic acid is broad and involves changes in the transcription levels of genes involved in carbohydrate metabolism, membrane multidrug resistance (MDR) transporters, lipid and amino acid metabolism, cell wall biosynthesis, protein folding, and nucleic acid processing [21, 22, 35]. Among the responsive genes, *TPO2* and *TPO3* are upregulated by Haa1 upon acetic acid stress [4, 21, 22, 35].

Aqr1, Tpo2, and Tpo3 are multidrug resistance (MDR) transporters that belong to the major facilitator superfamily (MFS). They are members of the H^+^ antiporter family 1 (DHA1) which are membrane proteins with 12 predicted transmembrane segments (TMS). Aqr1 was reported to confer resistance to C_2_-C_6_ short-chain monocarboxylic acids (e.g., acetic acid, propionic acid, butyric acid, and hexanoic acid) in *S. cerevisiae* [36]. An increased resistance against acetic acid, and also to the drugs flucytosine and clotrimazole, was observed in *Candida glabrata* upon *AQR1* expression [6]. Moreover, Aqr1 plays a role in the excretion of the amino acid alanine, aspartate, and glutamate [39]. Tpo2 and Tpo3, two paralogs in *S. cerevisiae*, are generally known as polyamine transporters which are specific for spermine [37]. Tpo2 and Tpo3, however, have a very broad substrate spectrum and also play a role in drug resistance and acid tolerance. The expression of *TPO3* in *C. glabrata* increases the tolerance to polyamines and various azole drugs, such as clotrimazole, tioconazole, and ketoconazole [5]. Deletion of *TPO2* or of *TPO3* decreases the tolerance to several acids, such as acetic acid, propionic acid, and benzoic acid [10]. Also acetaldehyde stress results in the improved expression of *TPO2* and *TPO3* regulated by Haa1 in *S. cerevisiae* [2].

The aforementioned work analyzed the impact of individual transporters in acid tolerance. In this work, the role of Aqr1, Tpo2 and Tpo3 was investigated through individual, double and triple gene deletion analysis to uncover their combined roles in acetic acid tolerance. Our data show that Tpo2 and to a lesser extent Tpo3 plays a key role in acetic acid stress and acetate efflux. Deletion of these genes is accompanied with increased cellular levels of acetate resulting in an altered gene transcription favoring ethanol production, and a downregulation of acetate metabolic genes.

## Results

### Acetic acid tolerance of putative acetate transporter deletion mutants

**Fig. 1:**
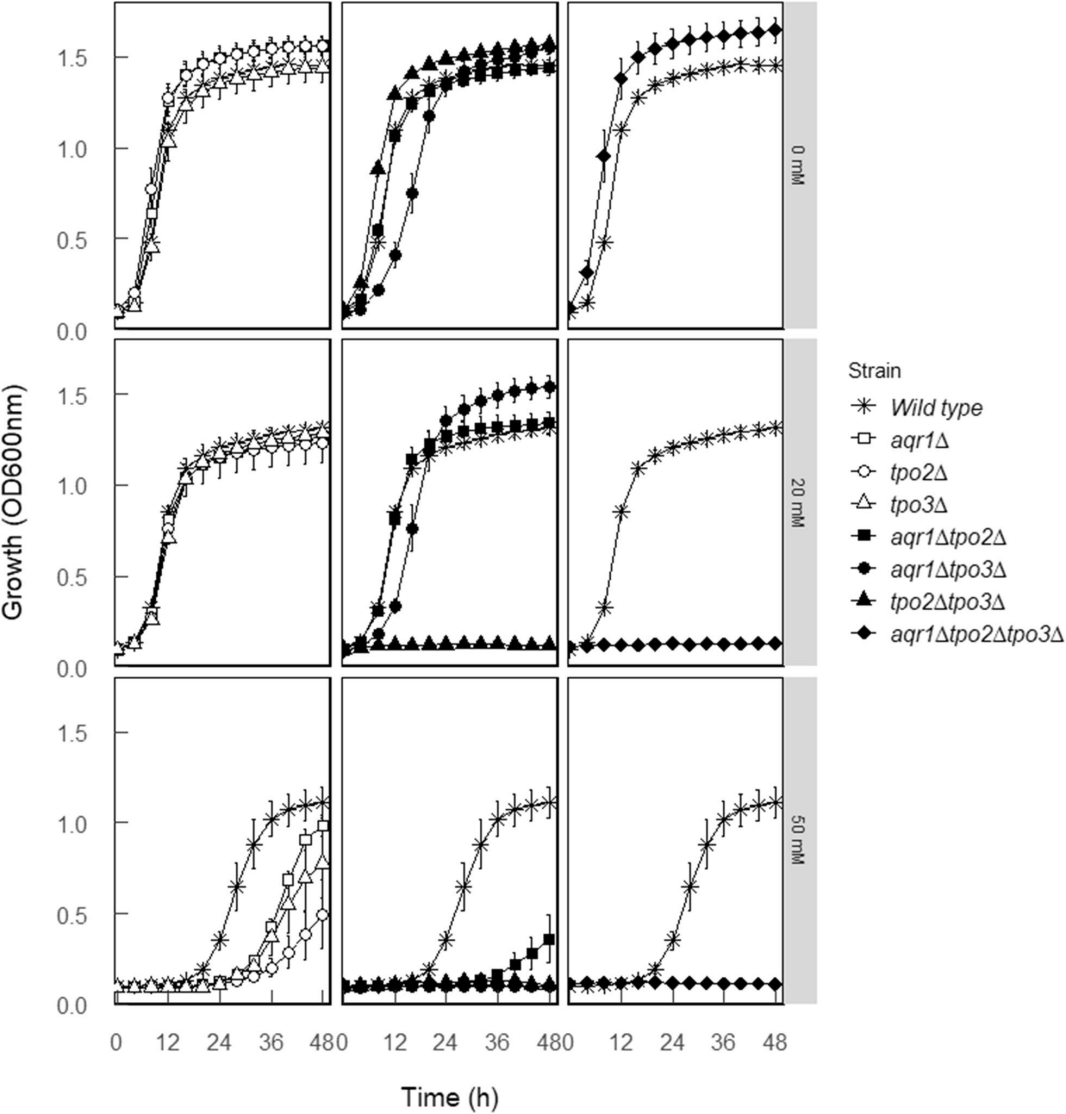
Growth of the *S. cerevisiae* CEN.PK 2-1D (wild type) and the derived acetate transporter deletion mutants, in 96-well microtiter plates in mineral medium complemented without or with 20 or 50 mM acetic acid at an initial pH value of 4. The left panel represents the comparison of growth curve between wild type and acetate transporter single deletion mutants, the middle panel represents the comparison of growth curve between wild type and acetate transporter double-deletion mutants, and the right panel represents the comparison of growth curve between wild type and acetate transporter triple deletion mutants. The data are the average of two biological replicates and two technical replicates

To analyze the combined impact of the putative acetic acid transporters Aqr1, Tpo2, and Tpo3 on acetic acid tolerance, individual, double, and triple gene deletion mutants were constructed using the CRISPR/Cas9 technique. Single gene deletions have been studied before [10, 36], but have not previously been combined to yield double and triple deletion strains. This allows us to resolve the combined role of this network of transporters in acetic acid tolerance. Therefore, acetate transporter single (*aqr1∆*, *tpo2∆*, and *tpo3∆*), double (*aqr1∆tpo2∆*, *aqr1∆tpo3∆*, and *tpo2∆tpo3∆*) and triple (*aqr1∆tpo2∆tpo3∆*) deletions were generated. Next, cells were grown on mineral medium with 2% (w/v) glucose and in the presence or absence of acetic acid at pH 4 (Fig. 1). In the absence of added acetic acid, the wild type and single transporter deletion strains showed identical growth and no apparent growth defect. Slightly increased maximal growth levels were noted with the *tpo2∆tpo3∆* mutant and the *aqr1∆tpo2∆tpo3∆* mutant, whereas the *aqr1∆tpo3∆* mutant showed a reduced growth rate. When cells were exposed to low levels of acetic acid (20 mM), growth of the wild type and all single acetate transporter deletion mutants was not affected, nor was growth of the *aqr1∆tpo2∆* mutant further affected. In contrast, the *tpo2∆tpo3∆* and *aqr1∆tpo2∆tpo3∆* mutants were unable to grow in the presence of 20 mM acetic acid (Fig. 1). At higher acetic acid concentration (50 mM), growth defects became apparent for nearly all strains but to varying degrees. Since the mutants with both *TPO2* and *TPO3* deletion were already unable to grow at low concentrations of acetic acid, it appears that Tpo2 and Tpo3 combined play an important role in acetic acid tolerance.

**Fig. 2:**
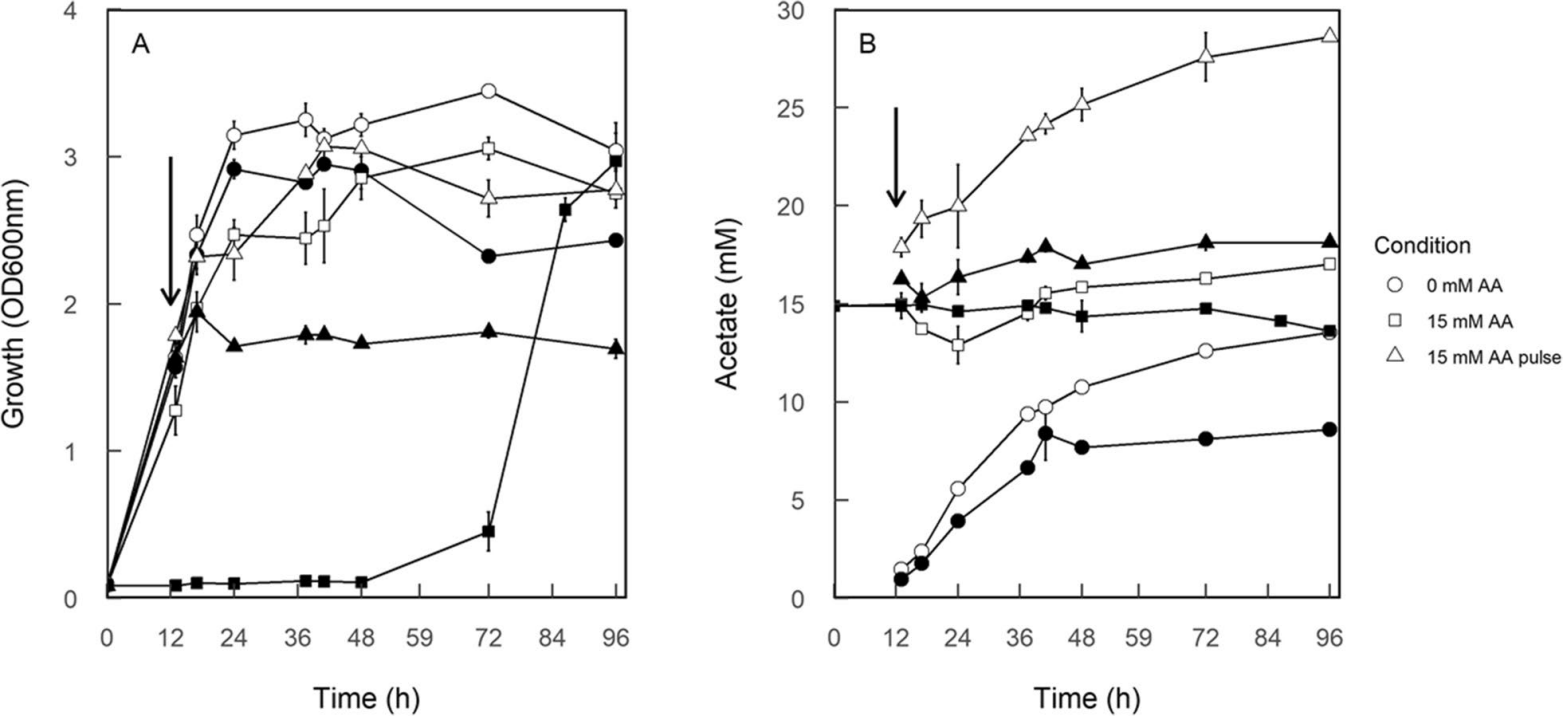
Growth profile (**A**) and extracellular acetate concentration (**B**) of the *S. cerevisiae* CEN.PK 2-1D (wild type shown in open symbols) and *tpo2∆tpo3∆* mutant (filled symbols) grown in shaking flasks containing 25 ml mineral medium at an initial pH value of 4 with (□) and without 15 mM acetic acid (○) or upon a 15 mM acetic acid pulse (∆) which 15 mM acetic acid was added during exponential phase indicated by arrow. The data are the average of two biological replicates

To further define the effects of the *TPO2* and *TPO3* double-deletion, growth profiles and acetate production were compared between the wild type and *tpo2∆tpo3∆* strain grown in shaken flasks. The lag phase of *tpo2∆tpo3∆* was extended to nearly 72 h in the presence of 15 mM acetic acid, while the wild type showed no significant lag phase extension (Fig. 2A). Addition of 15 mM acetic acid to the medium during exponential phase resulted in a growth arrest both for the wild type and *tpo2∆tpo3∆* after 3 h. However, the wild type resumed growth after 7 h, while *tpo2∆tpo3∆* remained in growth arrest for more than 4 days. Acetate production by the *tpo2∆tpo3∆* strain was always lower than that of the wild type (Fig. 2B). Following the acetic acid pulse stress, growth recovery by the wild type was not accompanied with net consumption of acetic acid. Rather, the extracellular acetic acid levels increased. Taken together, the deletion of *TPO2* and *TPO3* severely reduces the tolerance of *S. cerevisiae* towards acetic acid and its ability to recover from acetic acid stress.

### Expression of the putative acetate transporters in the various mutants

**Fig. 3:**
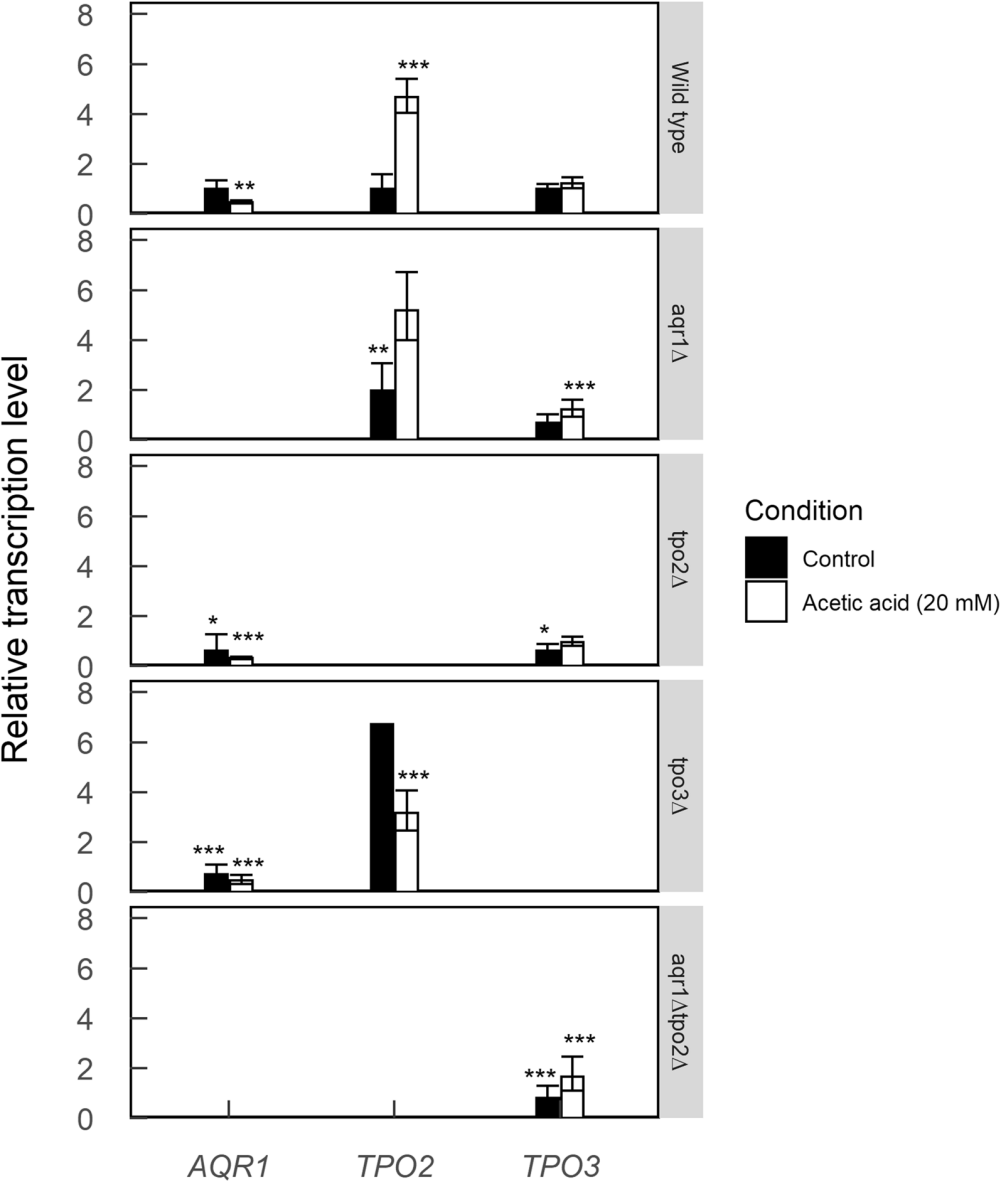
Transcription levels of acetate transporters in *S. cerevisiae* CEN.PK 2-1D (wild type) and mutants grown on glucose in the absence and presence of 20 mM acetic acid. All strains are grown to the exponential phase before RNA was extracted. The pH was set at 4.0. *ACT1* was used as internal control and the relative transcription represents the level of gene transcription normalized to the wild type transcription under the control cultivation conditions. Data are shown as average and standard deviation of biological duplicate. Statistical differences were performed by R programing. One-way ANOVA analysis and Tukey’s honest significant difference test were used to analyze the statistical difference between mutants and wild type under the same growth condition. *P ≤ 0.05, **P ≤ 0.01, ***P ≤ 0.001

Since the severe growth defects at lower acetic acid concentration were only observed with cells harboring both the *TPO2* and *TPO3* deletion, and not in cells with individual gene deletions, the transcriptional levels of *AQR1*, *TPO2*, and *TPO3* were determined to examine if the expression of the remaining exporters changes in the single deletion strains. Cells were grown in mineral medium with and without 20 mM acetic acid, and the transcription of the aforementioned genes was determined by RT-PCR using the *ACT1* gene as an internal reference from early exponential grown cells (Fig. 3). Transcriptional levels were normalized relative to that of the same gene in the wild type grown in the absence of acetic acid. With the wild type, growth in the presence of 20 mM acetic acid significantly increased the transcription of *TPO2* fivefold. Likewise, increased levels of *TPO2* transcription were also noted in the *aqr1∆* (2.0 ± 0.6) and *tpo3∆* (6.7 ± 0.5) mutants grown in the absence of acetic acid. *TPO2* transcriptional levels increased further (5.2 ± 0.4) in the *aqr1∆* strain but decreased (3.2 ± 0.4) in the *tpo3∆* strain when grown in the presence of 20 mM acetic acid. In the presence of 20 mM acetic acid, a decrease in *AQR1* transcription was noted in the wild type, *tpo2∆*, and *tpo3∆* strains. Further, the transcription of *TPO3* was slightly increased in the *aqr1∆* and *tpo2∆* strains, and in the *aqr1∆tpo2∆* strain when grown in the presence of acetic acid. In the *tpo2∆* strain, no significant changes in transcription of *TPO3* and *AQR1* were noted. Because of the significant growth defects of the strains lacking both *TPO2* and *TPO3* in the presence of acetic acid, *AQR1* levels were not determined in those cells. Since a major upregulation of *TPO2* occurred in the individual *aqr1∆* and *tpo3∆* mutants when cells were grown in the presence of acetic acid, the elevated levels of Tpo2 in these cells likely contributes to the remaining acetic acid tolerance and hence cause a weaker phenotype.

**Fig. 4:**
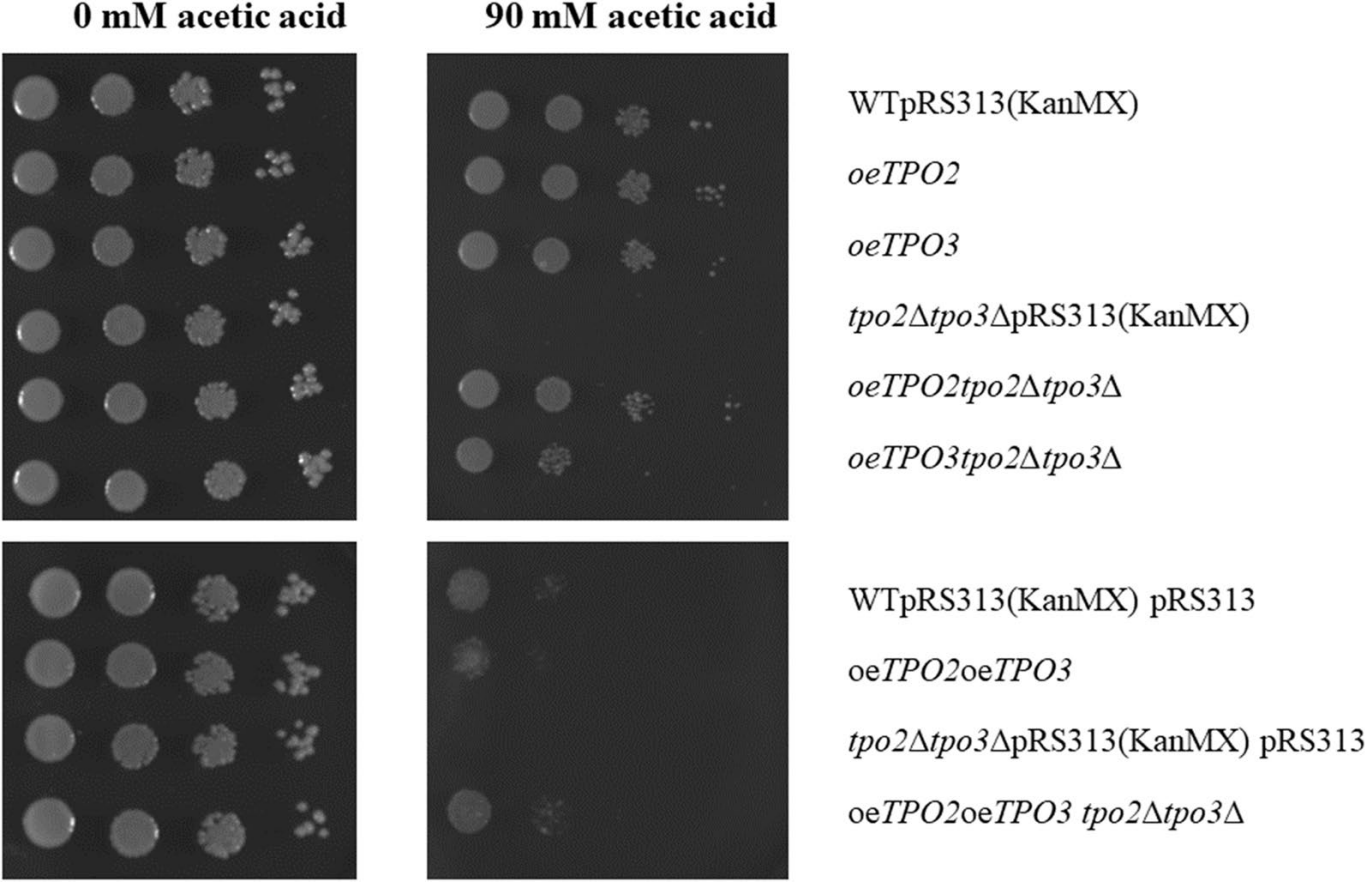
Acetate resistance of wild type and *∆tpo2∆tpo3∆* overexpressing *TPO2* and/or *TPO3*. Serial dilutions of 1 to 10^–3^ OD cell cultures were spotted on the plates with or without 90 mM acetate (pH 4). Images were taken after 3 days of growth

Since the above data indicate that Tpo2 and Tpo3 are the main acetic acid exporters, their ability to restore acetic acid tolerance was tested in the *tpo2∆tpo3∆* strain. Herein, the respective genes were cloned into the expression vector pRS313-P7T7 containing the truncated promoter and terminator of HXT7 [26]. The resulting plasmids were transformed into the wild type and the *tpo2∆tpo3∆* strain for overexpression of *TPO2* and/or *TPO3*. Spot assays were employed to compare the impact of *TPO2* and TPO3 overexpression on acetate resistance (Fig. 4). In the *tpo2∆tpo3∆* strain, Tpo2 or Tpo3 overexpression restored the acetate tolerance to wild type levels, with *TPO2* overexpression being more effective than *TPO3* overexpression. Combined *TPO2* and *TPO3* overexpression did not improve the tolerance to acetate beyond that observed for the wild type, suggesting that high level tolerance depends on other cellular processes.

### Accumulation of intracellular acetate

**Fig. 5:**
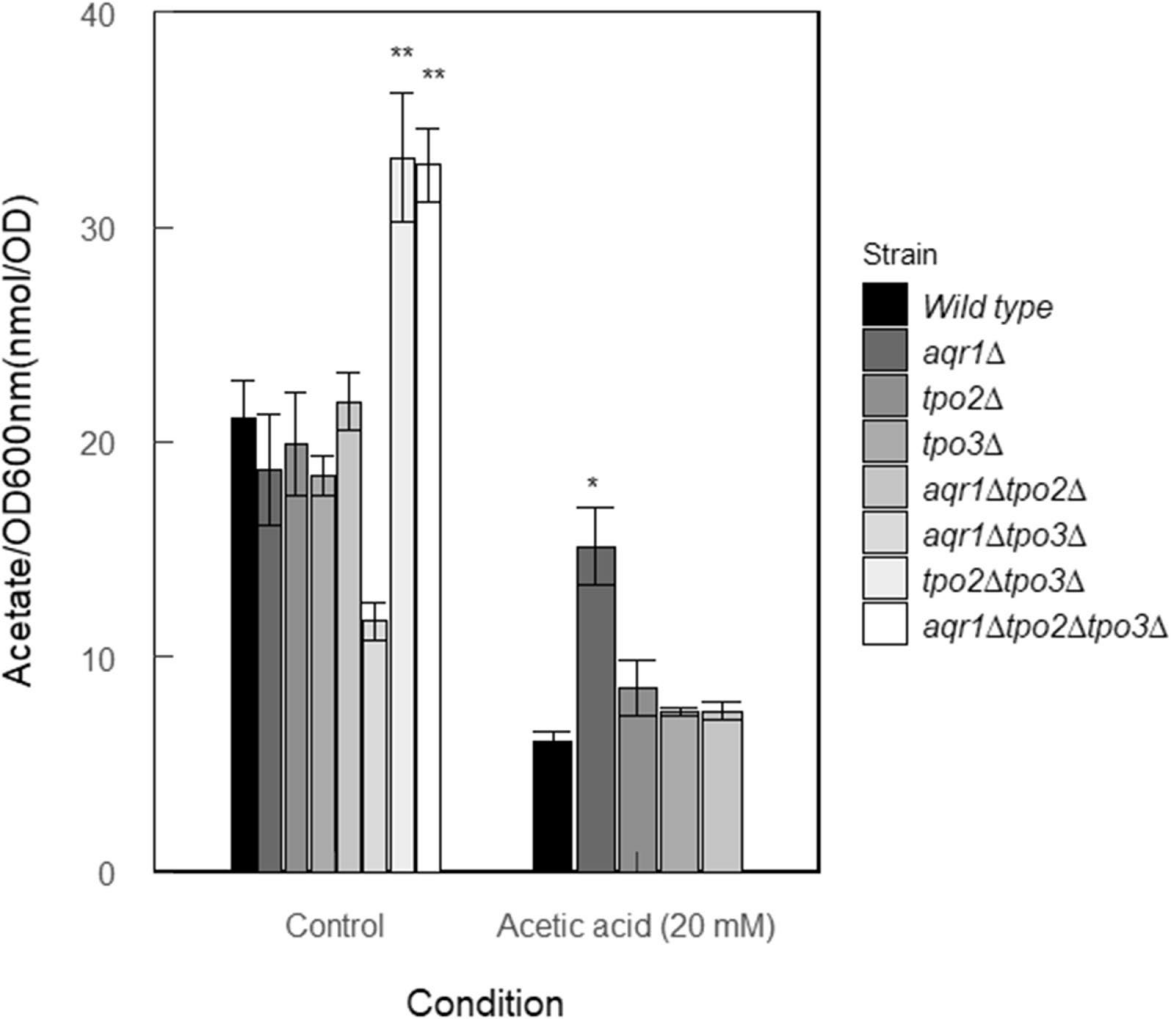
Intracellular acetate accumulation (in nmol/OD) in *S. cerevisiae* CEN.PK 2-1D (wild type) and mutant cells grown to exponential phase in mineral medium (pH 4) supplemented without and with 20 mM acetic acid. Data are shown as average and standard deviation of two biological replicates and two technical replicates. Statistical differences were performed by R programing. One-way ANOVA analysis and Tukey’s honest significant difference test were used to analyze the statistical difference between mutants and wild type under the same growth condition. *P ≤ 0.05, **P ≤ 0.01

Next, the intracellular levels of acetic acid in the various deletion mutants were determined. Herein, cells grown in the absence of acetic acid were collected from the mid-exponential phase, and the cellular metabolites were extracted with cold methanol. The mutants harboring both the *TPO2* and *TPO3* deletion, showed a significantly higher level of intracellular acetate than the control cells and the other deletion mutants (Fig. 5). This is consistent with the notion that acetate formed during sugar metabolism will be less efficiently secreted in these cells, although altered metabolism might also contribute to this phenomenon. Acetate levels were also determined in cells grown in the presence of 20 mM acetic acid. It should be noted that cells harboring both the *TPO2* and *TPO3* deletion showed a severe growth defect and therefore could not be used in these studies. In the other strains, levels of acetate were found to be much lower as compared to the growth conditions without extracellular acetic acid. Only in the *aqr1∆* mutant, the intracellular level of acetic acid was slightly lower than that in the control cells. In the presence of 20 mM acetic acid, no major changes in extracellular acetic acid levels were noted in the wild type, the single deletion mutants and the *aqr1∆tpo2∆* mutant (Additional file 1: Fig. S1).

### Acetate efflux analysis

**Fig. 6:**
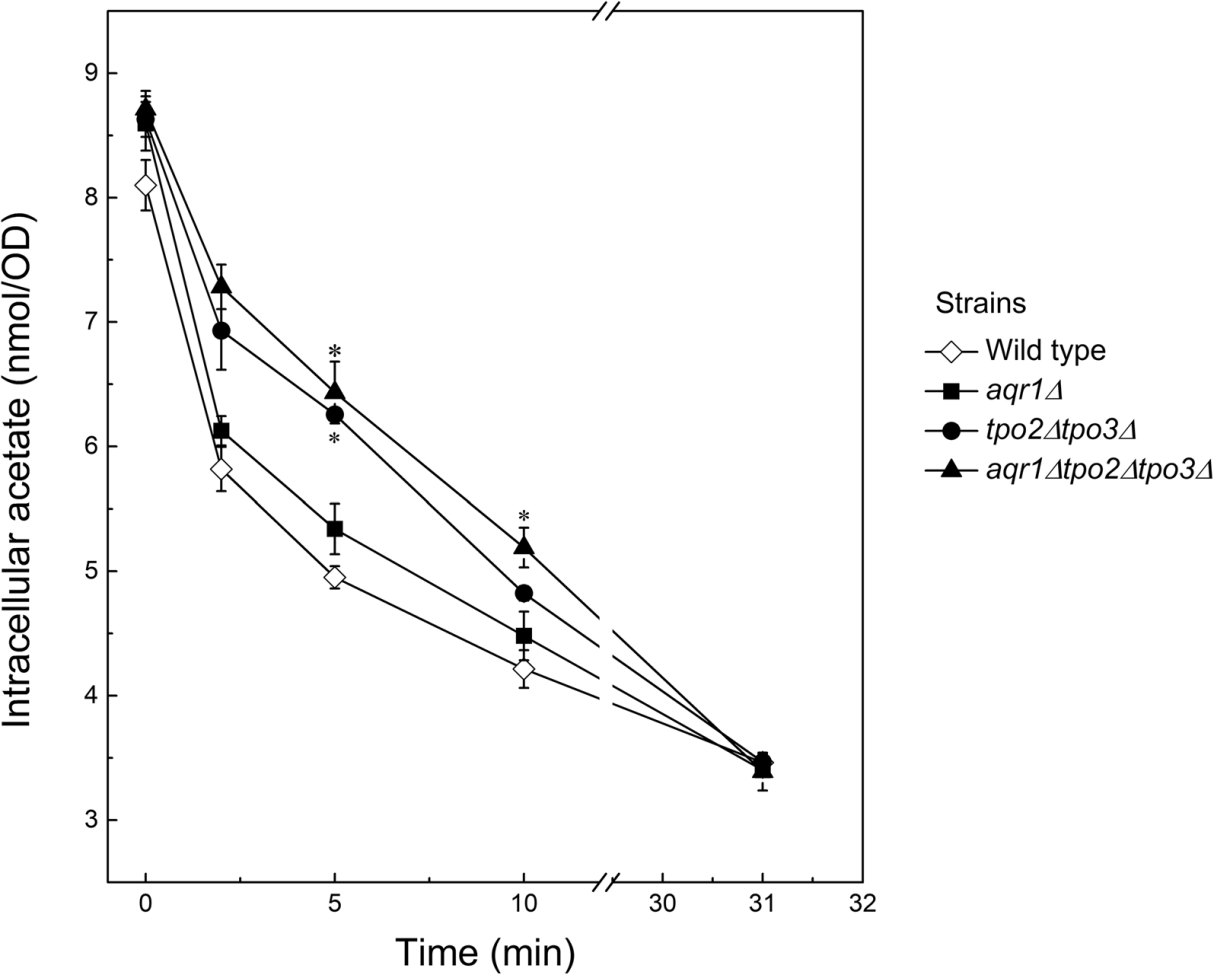
Acetate secretion by *S. cerevisiae* CEN.PK 2-1D (wild type) (◇) and *aqr1∆* (◼), *tpo2∆tpo3∆* (●) and *aqr1∆tpo2∆tpo3∆* (▲) mutants. Cells were loaded with 50 mM [1-^14^C-] acetate for 15 min and subsequently diluted into a medium with acetate, and the intracellular [1-^14^C] acetate was measured in time by filtration as described in the Materials and methods section. Data are shown as average and standard deviation of biological duplicate and expressed in nmol acetate/OD_600_. One-way ANOVA analysis and Tukey’s honest significant difference test were used to analyze the statistical difference between mutants and wild type at the same time point. *P ≤ 0.05

To determine if the reduced tolerance against acetic acid in the deletion strains is due to reduced secretion, efflux of acetate was measured employing cells grown in the absence of acetic acid. Cells were equilibrated for 15 min with 50 mM [1-^14^C] acetic acid yielding initially loading levels of 8.10 ± 0.29, 8.60 ± 0.31, 8.63 ± 0.20, and 8.71 ± 0.21 nmol of acetic acid/OD_600_ for the wild type, *aqr1∆*, *tpo2∆tpo3∆*, and *aqr1∆tpo2∆tpo3∆* strains, respectively. Next, the efflux of acetic acid was measured by diluting the cells more than 20-fold into a medium without acetic acid. Whereas the rate of acetic acid efflux was almost similar for the wild type and *aqr1∆* mutant, slower efflux was observed in the strains lacking both the *TPO2* and *TPO3* genes (Fig. 6). After long term incubation (> 30 min), the levels of remaining [1-^14^C] acetate were identical for all strains (Fig. 6). These data demonstrate that deletion of the Tpo2 and Tpo3 transporters indeed results in a reduced efflux of acetate.

### Sugar fermentation of deletion strains

Next, the various deletion strains were grown both anaerobically and aerobically on glucose, and the ethanol and acetate yield on glucose was determined. Increased levels of ethanol production were observed for the deletion strains relative to the wild type both under anaerobic and aerobic conditions (Table 1). Moreover, statistically significant differences of ethanol production were found in all mutants under aerobic condition, and in *aqr1∆tpo2∆*, the *tpo2∆tpo3∆* and *aqr1∆tpo2∆tpo3∆* mutants under anaerobic condition. In the anaerobic fermentation, this effect was most pronounced for the *aqr1∆tpo2∆*, the *tpo2∆tpo3∆* and *aqr1∆tpo2∆tpo3∆* mutants. The levels of acetic acid production remained mostly unaffected in aerobic and anaerobic fermentation or were slightly decreased in the *tpo2∆tpo3∆* and *aqr1∆tpo2∆tpo3∆* mutants under anaerobic growth conditions (Table 1). These data suggest an altered ethanol metabolism, in particular in strains lacking both *TPO2* and *TPO3*.

**Table 1:** Ethanol and acetate yield by wild type and mutant cells grown aerobically (9.5 h) or anaerobically (48 h) on glucose

Strain	Aerobic fermentation		Anaerobic fermentation	
	Ethanol yield g/g glucose consumption	Acetate yield g/g glucose consumption	Ethanol yield g/g glucose consumption	Acetate yield g/g glucose consumption
Wild type	0.371 ± 0.008	0.019 ± 0.003	0.386 ± 0.011	0.020 ± 0.001
*aqr1∆*	0.442 ± 0.019^*^	0.021 ± 0.004	0.402 ± 0.001	0.021 ± 0.001
*tpo2∆*	0.478 ± 0.024^**^	0.021 ± 0.002	0.396 ± 0.012	0.019 ± 0.001
*tpo3∆*	0.458 ± 0.004^**^	0.020 ± 0.001	0.410 ± 0.010	0.020 ± 0.001
*aqr1∆tpo2∆*	0.476 ± 0.006^**^	0.019 ± 0.001	0.431 ± 0.005^**^	0.022 ± 0.004
*tpo2∆tpo3∆*	0.454 ± 0.006^**^	0.019 ± 0.001	0.434 ± 0.002^**^	0.018 ± 0.001
*aqr1∆tpo2∆tpo3∆*	0.452 ± 0.012^*^	0.020 ± 0.001	0.434 ± 0.001^**^	0.018 ± 0.001

Statistical differences were performed by R programing

One-way ANOVA analysis and Tukey’s honest significant difference test were used to analyze the statistical difference between mutants and wild type under the same growth condition

*P ≤ 0.05, **P ≤ 0.01

### Transcription of genes related to acetic acid metabolism

**Fig. 7:**
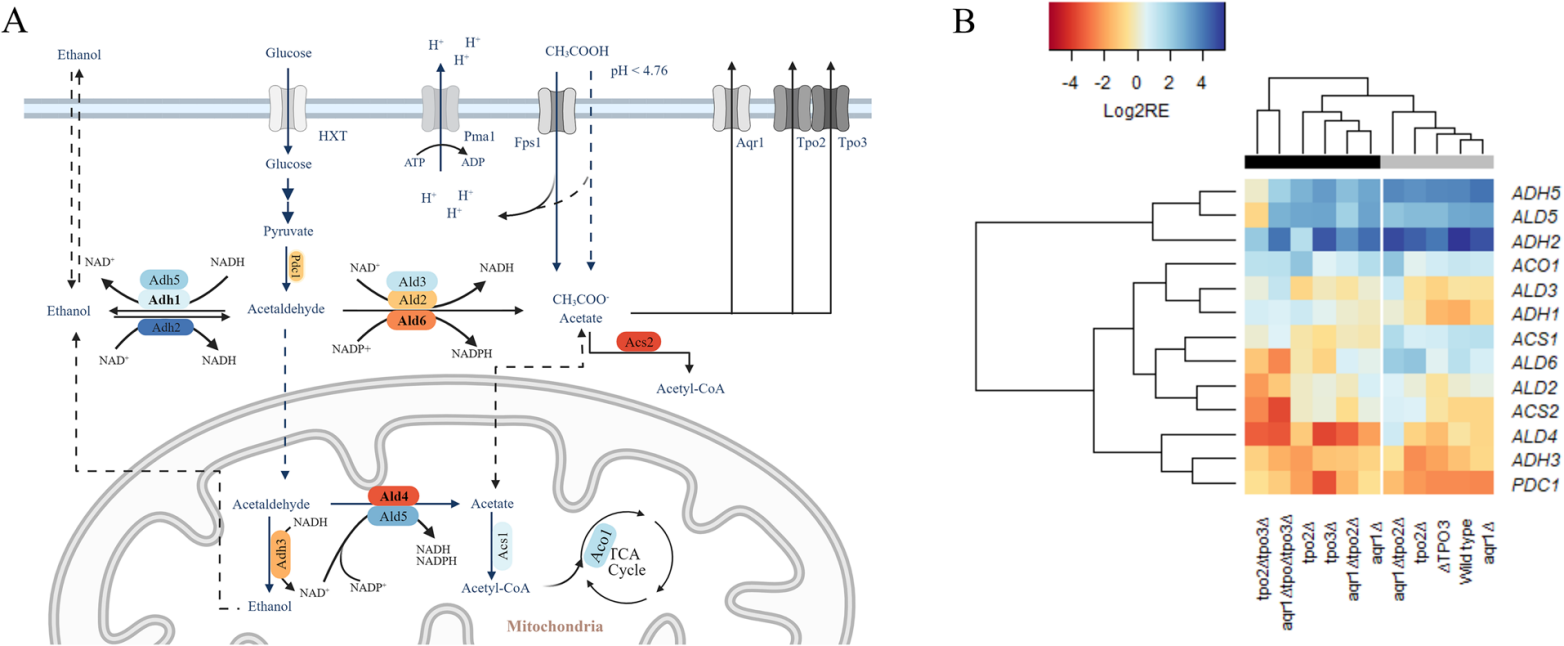
Metabolic pathway of ethanol and acetate metabolism in *S. cerevisiae* (**A**). The enzymes related to ethanol biosynthesis and acetate formation/degradation are: Pdc1, pyruvate decarboxylase; Adh1-5, alcohol dehydrogenase; Ald2-6, acetaldehyde dehydrogenase and Acs1-2, acetyl coenzyme A (CoA) synthase. Major enzymes are shown in bold. The colors of genes represented the log2 relative transcription of genes of *aqr1∆tpo2∆tpo3∆* in the absence of acetic acid, which are corresponding to the heatmap. Heatmap (**B**) representation of the log2 relative transcription of genes involved in ethanol and acetate metabolism in *S. cerevisiae* CEN.PK 2-1D (wild type) and acetate transporter deletion mutants in mineral medium without (black bar) or with 20 mM acetic acid (grey bar). The plotted relative transcription was normalized to the wild type levels in the absence of acetic acid. The color scale indicates the relative transcription levels with red downregulation and blue upregulation. Data are shown as the average of biological duplicates

In *S. cerevisiae*, pyruvate decarboxylase (Pdc1) converts pyruvate into acetaldehyde, which is further converted to ethanol by alcohol dehydrogenase (Adh1, Adh3, Adh4 and Adh5) or is oxidized to acetate by acetaldehyde dehydrogenase (Ald2, Ald3, Ald4, Ald5, Ald6) (Fig. 7A). The cytosolic enzymes Ald2, Ald3 and mostly prominent Ald6, catalyze acetate formation from glucose, while the mitochondrial enzymes Ald5 and in particular Ald4 are expressed during growth on ethanol to connect ethanol to primary metabolism. Ald2 and Ald3 play a limited role in this process as their genes are expressed by excessive levels of acetaldehyde [1] and during osmotic stress and glucose exhaustion [25, 32]. Adh4 and Adh5 are not involved in the reduction of acetaldehyde to ethanol [7].

To examine how ethanol and acetic acid metabolism are affected in the various deletion strains, the transcriptional levels of the genes involved in these pathways (Fig. 7A) were determined by RT-PCR. For this purpose, RNA was extracted from the wild type and deletion strains grown to early exponential phase in mineral medium with or without 20 mM acetic acid. The transcription of all analyzed genes was normalized relative to that in the wild type using *ACT1* as reference gene. With most of the deletion strains, upregulation was observed for the *ADH2*, *ADH5*, *ALD5*, and *ACO1* genes while *ALD2*, *ALD4*, *ALD6*, *ADH3*, *ACS2*, and *PDC1* were downregulated in the absence of acetic acid (Fig. 7B). In particular, the downregulation of the major acetaldehyde dehydrogenase Ald4 and Ald6, and the upregulation of alcohol dehydrogenases Adh1 in the mutants are in line with the increased levels of ethanol production. In the transcriptional response, the two strains lacking both *TPO2* and *TPO3* clustered together and show the strongest responses. These strains show remarkably reduced transcriptional levels (fold-change) of *ALD4* (9.4 ± 0.3 and 10.6 ± 0.4 in *tpo2∆tpo3∆* and *aqr1∆tpo2∆tpo3∆* strains, respectively)*, ALD6* (2.3 ± 0.2 and 5.5 ± 0.6) and *ACS2* (5.5 ± 0.3 and 12.2 ± 0.5). Overall, these data suggest that a reduction in the capacity to secreted acetic acid is accompanied with an altered metabolic gene transcription pattern that favors conversion of acetaldehyde into ethanol, hence preventing further accumulation of intracellular acetic acid. Interestingly, when the wild type and deletion mutants were challenged with 20 mM acetic acid, also higher levels of *ADH2, ACO1*, *ALD5* and *ADH5* and reduced levels of *PDC1* and *ADH3* were observed. Most notably, the transcriptional levels of *ACS1* and *ALD6* changed from downregulation to upregulation while *ADH1* transcripts were downregulated in the presence of acetic acid, which likely limits the accumulation of intracellular acetate by shuttling the acetic acid into the TCA cycle.

## Discussion

In agreement with previous studies [10, 36], *AQR1*, *TPO2*, and *TPO3* single gene deletion mutants exhibited a reduced tolerance to a high concentration of acetic acid. A previous study noted a detrimental effect of a *TPO3* deletion on the growth of the BY4741 strain [10]. Our double and triple deletion mutant studies in the CEN.PK strain confirm a major role for Tpo3, but also suggest a more prominent role of Tpo2. Although the *aqr1∆ tpo3∆* deletion strain already showed some reduced growth phenotype in the absence of acetic acid, a much more pronounced growth defect at low acetic acid concentrations was evident with the strains lacking both *TPO2* and *TPO3*. Interestingly, *TPO2* was strongly upregulated in cells exposed to acetic acid except in the *tpo3∆* mutant. However, in the absence of acetic acid, *TPO2* was also upregulated in the *tpo3∆* mutant as well as in *aqr1∆* cells. The elevated levels of Tpo2 likely contribute to the remaining acetic acid tolerance of the latter deletion strains, and thus will partially compensate for the reduced acetic acid tolerance observed in these deletion strains. A functional link between expression and tolerance was demonstrated by the expression of Tpo2 (or Tpo3) in the *tpo2∆ tpo3∆* double mutant which restored the acetic acid tolerance to wild type levels.

Tpo3 is a paralog of Tpo2, and both are regulated by the transcription factor Haa1 which plays a crucial role in response to acetic acid [10, 14, 21–23]. The intracellular acetate pool is considered to be a key factor to activate the Haa1 and Hog1 MAP kinase [21, 22, 29]. A highly sensitive field-effect transistor (FET)-type biosensor based on carbon nanofibers (CNF-FET) was employed to explore the weak acid anion binding site of Haa1 and it was demonstrated that acetate binds to a specific region of Haa1 subsequently activating Haa1 translocation [14]. That assay showed that a concentration of acetic acid as low as 1 nM already generates almost the same response as 100 nM which implies that very low concentrations of intracellular acetic acid already suffice to activate Haa1. Considering a cell volume of 15.9–42.4 um^3^ [38], the intracellular levels of acetic acid observed in this study are in the millimolar range and this would by far exceed the threshold of Haa1 activation irrespective of the presence or absence of acetic acid in the medium. The continuously increasing acetic acid concentration found in the wild type when acetic acid is added during exponential phase (Fig. 2B) makes it unlikely that intracellular acetate is the only trigger factor, suggesting the involvement of other regulatory factors [8] in addition to Haa1.

Metabolite and gene transcription analysis indicate an increased flux of acetaldehyde towards ethanol in the deletion strains. For instance, the *tpo2∆tpo3∆* and *aqr1∆tpo2∆tpo3∆* mutants show a higher ethanol yield in the absence of acetic acid, but these strains also accumulate acetate to higher levels as compared to the wild type. This can be attributed to a higher expression level of *ADH1* and lower transcript levels of the cytosolic *ALD6* and mitochondrial *ALD4*. *ADH1*, *ALD6* and *ACS1* which are the key genes whose transcription changes in the presence of extracellular acetic acid. Acs1 is crucial for the utilization of nonfermentable carbon sources and is downregulated when cells are grown on glucose or ethanol [30]). On the other hand, in the presence of additive acetic acid, an increased transcription level of *ACS1* is observed suggesting consumption of excess acetate via TCA cycle [34]. The transcriptional data show a clear distinction between *ADH5*, *ALD5* and *ADH3*, *ALD4* in the wild type and all mutants irrespective whether the mutants were challenged with acetic acid or not. Notably, in the mutants, the transcript levels of *ADH5* and *ALD5* increased while those of *ADH3* and *ALD4* decreased. Adh3 acts in the mitochondria and uses NADH as cofactor to generate ethanol. It is constitutively expressed during diauxic growth, i.e., during the glucose and ethanol utilization phases and is dispensable for the reduction of acetaldehyde to ethanol [7]. However, Adh3 participates in the ethanol–acetaldehyde shuttle to transport mitochondrial NADH to the cytosol in order to supplement the NADH pool depleted by other Adh enzymes [3]. The downregulation of mitochondrial *ALD4*, which uses NAD^+^ as the co-enzyme [25] and that converts acetaldehyde to acetate, will lead to a reduced capacity to generate NADH, which in turn may affect the transcriptional level of *ADH3*. Additionally, the mitochondrial Ald5 uses NAD^+^ or NADP^+^ as cofactor to convert acetaldehyde to acetate [25, 40]. The transcription of *ALD4* and *ALD5* depends on many factors, including the strain background, the culture medium, and the presence or absence of other alcohol dehydrogenase enzymes [32, 40]. For example, *ALD5* deletion reduces acetate production while *ALD4* deletion has no effect on acetate production. Whereas mutants devoid of *ALD4* and *ALD6* show defects when grown on glucose, deletion of both *ALD5* and *ALD6* has no effect on growth. Further, Ald5 is also involved in maintaining the mitochondrial electron transport chain [15, 28]. The multifunctional role of Ald5 renders it difficult to fully explain the potential impact of the upregulation of *ALD5* in the acetate transporter deletion strains upon acetic acid stress. However, the increased transcriptional levels of *ADH2* and *ACO1* and the decreased levels of *PDC1* both in the absence and presence of acetic acid indicate that the acetate transporter deletion mutants show a response reminiscent to acetic acid stress.

## Conclusions

In this study, deletion of the genes of the putative acetate exporters Aqr1, Tpo2, and Tpo3, both individually and in various combinations, was used to investigate the transporter-based mechanism of acetic acid tolerance. Growth assays and transcriptional analysis suggest that Tpo2, and to a lesser extent Tpo3, play a dominant role in acetic acid tolerance, acting as a major acetate efflux system. Deletion of both *TPO2* and *TPO3* caused a severe defect in acetate efflux and loss in acetic acid tolerance. Aqr1 fulfills only a minor role in these processes. In the absence of Tpo2 and Tpo3, a reduced efflux capacity is accompanied with an increased intracellular level of acetate. The deletion mutants showed an increased ethanol yield on glucose, and this can be correlated with an altered transcription of the major genes involved in ethanol and acetate metabolism most notably an increased transcription of *ADH1* and a reduced transcription of *ALD6* and *ALD4.* Taken together, this study provides a deeper insight into the transporter-based acetate tolerance mechanism and shows that the combination of Tpo2 and Tpo3 provides a major exporter-based tolerance mechanism towards high concentrations of acetate.

## Materials and methods

### Yeast strains

*Saccharomyces cerevisiae* CEN.PK 2-1D (MATalpha; his3∆1; leu2-3/112; ura3-52; trp1-289; MAL2-8c SUC2), a gift from DSM Bio-based Products and Services (Delft, The Netherlands), was used as the parental strain. All strains and plasmids used in this study are listed in Table 2 and Additional file 1: Table S1, respectively. A CRISPR/Cas9-based gene editing tool [19] was employed and yeast transformants were obtained using the LiAc-PEG protocol [33]. Oligonucleotides, used in CRISPR/Cas9 protocol, are listed in Additional file 1: Table S2.

**Table 2:** *S. cerevisiae* strains

Strain	Relevant genotype and/or characteristics	Source or reference
CEN.PK 2-1D	MATalpha; his3∆1; leu2-3/112; ura3-52; trp1-289; MAL2-8c SUC2	DSM, The Netherlands [9]
*aqr1*∆	CEN.PK 2-1D; aqr1∆	This study
*tpo2*∆	CEN.PK 2-1D; tpo2∆	This study
*tpo3*∆	CEN.PK 2-1D; tpo3∆	This study
*aqr1*∆*tpo2*∆	CEN.PK 2-1D; aqr1∆; tpo2∆	This study
*aqr1*∆*tpo3*∆	CEN.PK 2-1D; aqr1∆; tpo3∆	This study
*tpo2*∆*tpo3*∆	CEN.PK 2-1D; tpo2∆; tpo3∆	This study
*aqr1*∆*tpo2*∆*tpo3*∆	CEN.PK 2-1D; aqr1∆; tpo2∆; tpo3∆	This study
WTpRS313(KanMX)	CEN.PK 2-1D; pRS313-P7T7-KanMX	This study
*tpo2*∆*tpo3*∆pRS313(KanMX)	*tpo2*∆*tpo3*∆; pRS313-P7T7-KanMX	This study
oe*TPO2*	CEN.PK 2-1D; pRS313-P7T7-KanMX-TPO2	This study
oe*TPO3*	CEN.PK 2-1D; pRS313-P7T7-KanMX-TPO3	This study
oe*TPO2tpo2*∆*tpo3*∆	*tpo2*∆*tpo3*∆; pRS313-P7T7-KanMX-TPO2	This study
oe*TPO3tpo2*∆*tpo3*∆	*tpo2*∆*tpo3*∆; pRS313-P7T7-KanMX-TPO3	This study
WTpRS313(KanMX) pRS313	CEN.PK 2-1D; pRS313-P7T7-KanMX; pRS313-P7T7	This study
*tpo2*∆*tpo3*∆pRS313(KanMX) pRS313	*tpo2*∆*tpo3*∆; pRS313-P7T7-KanMX; pRS313-P7T7	This study
oe*TPO2*oe*TPO3*	CEN.PK 2-1D; pRS313-P7T7-TPO2; pRS313-P7T7-KanMX-TPO3	This study
oe*TPO2*oe*TPO3 tpo2*∆*tpo3*∆	*tpo2*∆*tpo3*∆; pRS313-P7T7-TPO2; pRS313-P7T7-KanMX-TPO3	This study

### Growth assay

Yeast strains were grown in 100-mL Erlenmeyer flasks containing 25 mL mineral medium (MM) with 2% w/v D-glucose and the required amino acids that were used as markers [16]. The composition of the mineral medium is as follows: K_2_SO_4_, 6.6 g/L; KH_2_PO_4_, 3 g/L; MgSO_4_∙7H_2_O, 0.5 g/L; urea (NH_2_CONH_2_), 2.3 g/L; vitamin solution, 1 ml/L; trace element solution, 1 ml/L. Urea and vitamin solution were filter-sterilized and added into the autoclaved medium. The composition of the vitamin solution is as follows: biotin (D-) (C_10_H_16_N_2_O_3_S), 0.05 g/L; Ca D (+) pantothenate (C_18_H_32_CaN_2_O_10_), 1.00 g/L; nicotinic acid (C_6_H_5_NO_2_), 1.00 g/L; myo-inositol (C_6_H_12_O_6_), 25.00 g/L; thiamine chloride hydrochloride (C_12_H_18_C_l2_N_4_OS.xH_2_O), 1.00 g/L; pyridoxal hydrochloride (C_8_H_12_CINO_3_), 1.00 g/L; p-aminobenzoic acid (C_7_H_7_NO_2_), 0.20 g/L; NaOH, 4.00 g/L. The composition of trace element solution is as follows: EDTA, 15.00 g/L; ZnSO_4_∙7H_2_O, 4.50 g/L; MnCl_2_∙2H_2_O, 0.84 g/L; CoCl_2_∙6H_2_O, 0.30 g/L; CuSO_4_∙5H_2_O; 0.30 g/L; Na_2_MoO_4_∙2H_2_O, 0.40 g/L; CaCl_2_∙H_2_O, 4.50 g/l; FeSO_4_∙7H_2_O, 3.00 g/L; H_2_BO_3_, 1.00 g/L; KI, 0.10 g/L. HCl and KOH were used to adjust medium pH to 4. Cultivations were at 30 °C with constant shaking at 200 rpm. Overnight cultures grown under the same conditions were used as inoculum to an OD_600 nm_ of 0.1. Cell density was followed by measuring the optical density at 600 nm using an UV–visible spectrophotometer (Novaspec PLUS). For growth under micro-anaerobic condition, yeast strains were grown in 100 mL bottle with lid containing 100 mL MM with 2% glucose at 200 rpm and 30 °C. All growth assays were performed in at least duplicates.

### RNA extraction and cDNA synthesis

RNA extraction from yeast cells was performed by glass-bead disruption employing the Trizol extraction protocol (Life Technologies, Bleiswijk, The Netherlands). Six OD units of yeast cells from the exponential phase were harvested and mixed with 0.2 mL glass beads (diameter 0.45 mm), 900 μL Trizol and 125 µL chloroform. Next, the cells were disrupted with a Fastprep FP120 (Thermo Savant) for 45 s at speed 6. Extracted total RNA (500 ng) was used for reverse transcription to synthesize cDNA by using the iScript cDNA synthesis Kit (Bio-rad, CA, USA).

### Real-time PCR

Real-time qPCR was carried out with the SensiFAST™ SYBR® Hi-ROX kit (Bioline, Meridian Bioscience, Waddinxveen, Netherlands) employing the iCYCLER iQ real-time PCR (Bio-Rad). The actin gene was used to normalize the targeted fold changes of gene transcript levels. Primers used in qPCR are shown in Additional file 1: Table S3, and all qPCR experiments were carried out in at least duplicates.

### Metabolite analysis

Glucose, acetic acid, and glycerol were analyzed by HPLC equipped with a refractive index detector (Shimadzu, Kyoto, Japan) using a Aminex HPX-87H column (Bio-Rad) at a temperature of 65 °C using a mobile phase of 0.005 N H_2_SO_4_ and a flow of 0.55 ml/min.

Intracellular acetate was determined as described [17, 31]. Cells (10 OD units) were collected in exponential phase and washed twice with ice-cold demineralized water to remove extracellular metabolites. The cell pellet was resuspended in 1 mL pre-cold (− 20 °C) 50% (v/v) methanol, samples were incubated at − 80 °C for 30 min, and thawed in an ice bath for 10 min. After centrifugation (13,000 *g*, 10 min, 4 °C), the supernatant was transferred to a new tube, and the cell pellets was subjected to a second-round of metabolite extraction. The combined supernatants were dried by vacuum centrifugation (Eppendorf concentrator plus) for 4–6 h. After resuspension in 200 μL demineralized water, samples were filtered through a 0.2 um PTFE 13 mm syringe filter (514-0068) and then stored at − 20 °C until analysis by HPLC.

### Acetic acid transport assays

The efflux of [1-^14^C] acetic acid was analyzed as reported before [10, 27] with minor modifications. Yeast cells in the mid-exponential phase were collected by centrifugation at 3000 rpm for 3 min at 20 °C and washed with mineral medium without carbon source. Cells (20 OD units) were subsequently resuspended in mineral medium without carbon source, at a final volume of 50 μL and incubated with 50 mM [1-^14^C] acetic acid (1.85 GBq, Campro Scientific GmbH, Veenendaal, Netherlands) for 15 min at 200 rpm and 30 °C. Next, 1150 μL of mineral medium without carbon source was added to start the acetate efflux. At various times, 200 μL of the cell suspension was collected and filtered through 0.45-μm HV membrane filters (Millipore, France). Filters were washed twice with 4 ml of an ice-cold lithium chloride solution and counted using a liquid scintillation counter (Perkin-Elmer, USA).

## Supplementary Information

**Additional file 1: Table S1. **Plasmids. **Table S2**. Oligonucleotides used in CRISPR/Cas9 protocol. **Table S3**. Oligonucleotides used in qPCR. **Figure S1.** Extracellular acetic acid concentration (in mM) in wild type and mutants in the presence of 20 mM acetic acid. Cells were grown to exponential phase and the acetate level in the fermentation broth was determined. Data are shown as average and standard deviation of biological duplicate.

## Data Availability

All data generated or analyzed during this study are included in this published article and its Additional files.
