# Supplementary material for: Combined roles of exporters in acetic acid tolerance in Saccharomyces cerevisiae
Source: Biotechnol Biofuels Bioprod. 2022 Jun 18;15:67. doi: 10.1186/s13068-022-02164-4 (PMC9206328; doi:10.1186/s13068-022-02164-4)
Supplement: Supplementary file 1 — Additional file 1: Table S1. Plasmids. Table S2. Oligonucleotides used in CRISPR/Cas9 protocol. Table S3. Oligonucleotides used in qPCR. Figure S1. Extracellular acetic acid concentration (in mM) in wild type and mutants in the presence of 20 mM acetic acid. Cells were grown to exponential phase and the acetate level in the fermentation broth was determined. Data are shown as average and standard deviation of biological duplicate. [file 13068_2022_2164_MOESM1_ESM.docx]

**Combined roles of exporters in acetic acid tolerance in *Saccharomyces cerevisiae***

Xiaohuan Zhang, Jeroen G. Nijland and Arnold J.M. Driessen

Molecular Microbiology, Groningen Biomolecular Sciences and Biotechnology, University of Groningen, Groningen, Netherlands

**Table S1.** Plasmids.

| Plasmids | Relevant genotype and/or characteristics | Source or reference |
| --- | --- | --- |
| p414-TEF1p-cas9-CYC1t | CEN6/ARS4 ampR pTEF1-cas9-tCYC1 | [1] |
| pMEL10 | 2μm ampR URA3 gRNA-CAN1.Y | This study |
| pMEL14 | 2μm ampR KlLEU2 gRNA-CAN1.Y | This study |
| pMEL17 | 2μm ampR TRP1 gRNA-CAN1.Y | This study |
| pRS313-P7T7 | pRS313 vector with pHXT7-tHXT7 cassette | [2] |
| pRS313-P7T7-KanMX | pRS313 vector with pHXT7-tHXT7 cassette; HIS selection marker was replaced with G418 | [2] |
| pRS313-P7T7-TPO2 | pRS313 vector with pHXT7-TPO2-tHXT7 cassette | This study |
| pRS313-P7T7-KanMX-TPO2 | pRS313 vector with pHXT7-TPO2-HXT7 cassette; HIS selection marker was replaced with G418 | This study |
| pRS313-P7T7-KanMX-TPO3 | pRS313 vector with pHXT7-TPO3-HXT7 cassette; HIS selection marker was replaced with G418 | This study |
|  |  |  |

**Table S2**. Oligonucleotides used in CRISPR/Cas9 protocol

| Gene | Target sequence (including PAM) |
| --- | --- |
| *AQR1* | ACCAGAACTGATTGCAATTGTGG |
| *TPO2* | CACGAGAGTTGGTTTTTGAGGGG |
| *TPO3* | TCAATTTCCGGATCTAGATCTGG |

**Table S3**. Oligonucleotides used in qPCR

| Name | Sequence (5′🡪3′) |
| --- | --- |
| *ACT1* F | GGATTCTGAGGTTGCTGCTTTGG |
| *ACT1* R | GAGCTTCATCACCAACGTAGGAG |
| *AQR1* F | ATCTTTTATTTGGTTGGAGT |
| *AQR1* R | CGTTGGGTACAAATCGACCAATAAC |
| *TPO2* F | TCCATGGTGGACGTTGAGGG |
| *TPO2* R | CTGGAACCAGCCCAGATGGTG |
| *TPO3* F | CCTACAGGTTTAGTTCCTGTCGC |
| *TPO3* R | CGAATTCAATTTCCGGATCTAGATC |
| *ADH1* F | GAGATGGATGGGACACCAAC |
| *ADH1* R | ACGAATCCCACGGTAAGTTG |
| *ADH2* F | GTGCCCACGGTATCATCAAT |
| *ADH2* R | CGTAAGAGCCGACAATGGAG |
| *ADH3* F | GACCACCGACTAATGGTAGC |
| *ADH3* R | CGACCGCTGATGCTATTCAA |
| *ADH5* F | ACGTTAAGGGCTGGAAAGTC |
| *ADH5* R | ATCGGCAGTTGCGTATTCTT |
| *ALD2* F | ACTGGCGAACCGATAACATC |
| *ALD2* R | GTCTCTAATGCGGCAAGTGT |
| *ALD3* F | TGGTTATGGTTCCGTTGTGG |
| *ALD3* R | GGAGACTTACCACCGCATTC |
| *ALD4* F | CCCCATTGTCCGCTTTGTAT |
| *ALD4* R | CTGCGGACTGGTAAATGTGT |
| *ALD5* F | CGGTGGTTGGGCAGATAAAA |
| *ALD5* R | GTGTTACCTGTAGCCAGAGC |
| *ALD6* F | GCAACCAACCGGTCTATTCA |
| *ALD6* R | CCCATTCAGTGTCGTGGAAA |
| *PCD1* F | TGGTAACGCCAACGAATTGA |
| *PCD1* R | GAGATGGATGGGACACCAAC |
| *ACS1* F | TCTGGTTCGCATCTGGTCAC |
| *ACS1* R | GATGGCCATGCAGCTTTGAC |
| *ACS2* F | GTCGGTGAACCAATCTCTCC |
| *ACS2* R | ACCAAAGAATGGCACGGTAG |
| *ACO1* F | GCCATTGCGGGTGATTTGAG |
| *ACO1* R | TGCAGGTGGAGCTTGGTAAG |

**
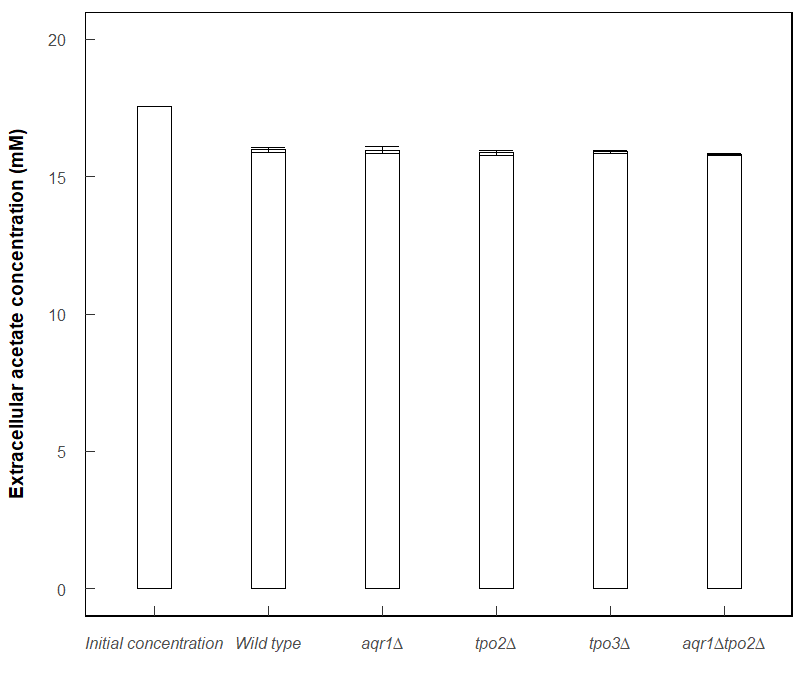
**

**Figure S1.** Extracellular acetic acid concentration (in mM) in wild type and mutants in the presence of 20 mM acetic acid. Cells were grown to exponential phase and the acetate level in the fermentation broth was determined. Data are shown as average and standard deviation of biological duplicate.

**Reference**

1. Nijland JG, Shin HY, Boender LGM, de Waal PP, Klaassen P, Driessen AJM: **Improved Xylose Metabolism by a CYC8 Mutant of Saccharomyces cerevisiae**. *Appl Environ Microbiol* 2017, **83**(11).

2. Nijland JG, Shin HY, de Jong RM, de Waal PP, Klaassen P, Driessen AJ: **Engineering of an endogenous hexose transporter into a specific D-xylose transporter facilitates glucose-xylose co-consumption in Saccharomyces cerevisiae**. *Biotechnol Biofuels* 2014, **7**(1):168.
